# Inhibition of NADPH oxidase activation reduces EAE-induced white matter damage in mice

**DOI:** 10.1186/s12974-015-0325-5

**Published:** 2015-05-28

**Authors:** Bo Young Choi, Jin Hee Kim, A Ra Kho, In Yeol Kim, Song Hee Lee, Bo Eun Lee, Eunhi Choi, Min Sohn, Mackenzie Stevenson, Tae Nyoung Chung, Tiina M Kauppinen, Sang Won Suh

**Affiliations:** Department of Physiology, College of Medicine, Hallym University, Chuncheon, South Korea; Chuncheon Sacred Heart Hospital, Department of Rehabilitation Medicine, College of Medicine, Hallym University, Chuncheon, South Korea; Department of Nursing, Inha University, Incheon, South Korea; CHA Bundang Medical Center, School of Medicine, CHA University, Kyunggi do, South Korea; Department of Pharmacology and Therapeutics, University of Manitoba, Winnipeg, Canada

**Keywords:** Experimental autoimmune encephalomyelitis, Multiple sclerosis, NADPH oxidase, Reactive oxygen species, Apocynin, Microglia

## Abstract

**Background:**

To evaluate the role of NADPH oxidase-mediated reactive oxygen species (ROS) production in multiple sclerosis pathogenesis, we examined the effects of apocynin, an NADPH oxidase assembly inhibitor, on experimental autoimmune encephalomyelitis (EAE).

**Methods:**

EAE was induced by immunization with myelin oligodendrocyte glycoprotein (MOG (35-55)) in C57BL/6 female mice. Three weeks after initial immunization, the mice were analyzed for demyelination, immune cell infiltration, and ROS production. Apocynin (30 mg/kg) was given orally once daily for the entire experimental course or after the typical onset of clinical symptom (15 days after first MOG injection).

**Results:**

Clinical signs of EAE first appeared on day 11 and reached a peak level on day 19 after the initial immunization. The daily clinical symptoms of EAE mice were profoundly reduced by apocynin. The apocynin-mediated inhibition of the clinical course of EAE was accompanied by suppression of demyelination, reduced infiltration by encephalitogenic immune cells including CD4, CD8, CD20, and F4/80-positive cells. Apocynin reduced MOG-induced pro-inflammatory cytokines in cultured microglia. Apocynin also remarkably inhibited EAE-associated ROS production and blood–brain barrier (BBB) disruption. Furthermore, the present study found that post-treatment with apocynin also reduced the clinical course of EAE and spinal cord demyelination.

**Conclusions:**

These results demonstrate that apocynin inhibits the clinical features and neuropathological changes associated with EAE. Therefore, the present study suggests that inhibition of NADPH oxidase activation by apocynin may have a high therapeutic potential for treatment of multiple sclerosis pathogenesis.

## Background

Multiple sclerosis (MS) is one of the most common neurological disorders and causes of disability in young adults [1] and is the most common inflammatory demyelinating disorder of the central nervous system (CNS). Hallmarks of MS include multifocal perivascular mononuclear cell infiltration in the CNS, oligodendrocyte loss, blood–brain barrier disruption, and demyelination. Axonal damage is also seen to occur after primary demyelination and can lead to permanent neurological deficits.

Reactive oxygen species (ROS) are involved in the pathogenesis of MS and experimental autoimmune encephalomyelitis (EAE) [2, 3]. Increased production of ROS following EAE is an important underlying cause of demyelination. Activated NADPH oxidases produce ROS that are released into the intra- or extracellular space and contribute to progressive demyelination. Due to the requirement of protein kinase activity for phosphorylation and translocation of NADPH oxidase subunits (e.g., p47^phox^) prior to its activation, this enzyme is directly regulated by plasma membrane receptors and signaling pathways [4]. Our previous studies have demonstrated that NADPH oxidase-derived ROS production modulates neuronal death in hypoglycemia, ischemia, epilepsy, and traumatic brain injury [5–9].

Apocynin is a compound isolated from the medicinal plant *Picrorhiza kurroa*. Apocynin inhibits NADPH oxidase activation in neutrophils [10, 11], macrophages, and systemic endothelia [12] through the inhibition of p47^phox^ subunit translocation. It has been reported that apocynin protects against global cerebral ischemia-induced oxidative stress and neuronal injury in the hippocampus [7, 13], and inhibiting ROS production by apocynin preserves blood–brain barrier [14]. Van der Goes et al. demonstrated that the phagocytosis of myelin by macrophages triggers the production of ROS, which play a crucial role in myelin phagocytosis. Using peritoneal macrophage culture, blocking ROS production with apocynin prevented the phagocytosis of myelin [15]. In addition, Van der Veen et al. has reported that neutrophil cytosolic factor 1 (NCF1, also known as p47^phox^) knockout mice are resistant to EAE [16]. However, the protective effects of apocynin have never been tested using the mouse model of EAE.

One of the best described and most commonly used animal models of MS is EAE. EAE is induced by immunization with myelin oligodendrocyte glycoprotein (MOG (35-55)) peptide [17, 18]. Using the MOG-induced EAE mouse model, we investigated if systemically delivered apocynin could suppress EAE disease progression. In the present study, we found that apocynin ameliorated the severity of the EAE in MOG-injected mice, which is accompanied by reduction of demyelination and ROS production, attenuation of microglial activation, inhibition of immune cell infiltration, and reduction of BBB disruption. These data establish inhibition of NADPH oxidase activation by apocynin as a potential therapeutic strategy in MS.

## Methods

### Induction of EAE

Animal use and relevant experimental procedures were approved by the Institutional Animal Care and Use Committee, Hallym University (Protocol # Hallym 2011-68). This manuscript was written in accordance with the ARRIVE (Animal Research: Reporting In Vivo Experiments) guidelines [19]. C57BL/6 female mice, aged 8 weeks, purchased from DBL (Chungcheongbuk, Korea) were housed in a temperature- and humidity-controlled environment and supplied with Purina diet (Purina, Gyeonggi, Korea) and water ad libitum. Mice were immunized on day 0 by subcutaneous injection of 200 μl of a mixture of recombinant MOG (35-55, 2 mg/ml) (AnaSpec, CA) and complete Freund’s adjuvant containing 400 μg of *Mycobacterium tuberculosis* H37RaA (Difco Laboratories, MI) according to the manufacturer’s instruction. Pertussis toxin (List Biological Laboratories, CA) was intraperitoneally administered at a dose of 400 ng on post-immunization days 0 and 2. A booster injection of MOG was given on day 7 after the initial immunization.

### Apocynin administration

Apocynin (Apo, Sigma, St Louis, MO) was dissolved with DMSO and diluted by a factor of 10 with saline (10 % DMSO). Apocynin was delivered by gavage once per day at a dose of 30 mg/kg/day from day 0 until the end of the experiment. Animals were divided into four groups to evaluate the pre-treatment effects of apocynin for short-term period: (1) sham without apocynin (DMSO only, *n* = 7), (2) sham with apocynin (apocynin only, *n* = 6), (3) EAE without apocynin (EAE + DMSO, *n* = 12), and (4) EAE with apocynin (EAE + apocynin, *n* = 12). Animals were divided into four groups to evaluate the pre-treatment effects of apocynin for long-term period: (1) sham without apocynin (DMSO only, *n* = 2), (2) sham with apocynin (apocynin only, *n* = 2), (3) EAE without apocynin (EAE + DMSO, *n* = 6), and (4) EAE with apocynin (EAE + apocynin, *n* = 6). To test the post-treatment effects of apocynin, apocynin was delivered 3 days after the typical onset of clinical symptoms (15 days after first MOG injection). Animals were divided into two groups: (1) EAE without apocynin (EAE + DMSO, *n* = 5) and (2) EAE with apocynin (EAE + apocynin, *n* = 5).

### Disease scoring

Behavior was scored daily for evaluation of clinical features of EAE according to the following criteria: 0, no deficit; 0.5, partial loss of tail tone or slightly abnormal gait; 1.0, complete tail paralysis or both partial loss of tail tone and mild hind limb weakness; 1.5, complete tail paralysis and mild hind limb weakness; 2.0, tail paralysis with moderate hind limb weakness (evidenced by frequent foot dropping between bars of cage top while walking); 2.5, no weight-bearing on hind limbs (dragging) but with some leg movement; 3.0, complete hind limb paralysis with no residual movement; 3.5, hind limb paralysis with mild weakness in forelimbs; 4.0, complete quadriplegia but with some movement of head; 4.5, moribund; and 5, dead [20]. General linear models (GLM) repeated measures were conducted to investigate differences of clinical scores changing over time among groups. The maximal clinical scores were also compared between vehicle- and apocynin-treated EAE animals.

### Histological evaluation of the spinal cord and brain

On day 21 after the initial immunization, mice were transcardially perfused with 4 % paraformaldehyde (PFA) under urethane anesthesia. The brain and spinal cord were removed and post-fixed in the same fixative. Paraffin-embedded sections at 5 μm were made after embedding in paraffin and then stained with luxol fast blue (LFB) to detect demyelination. Frozen sections at 30 μm were stained with cresyl violet to determine mononuclear cell infiltration. To quantify LFB staining results, five sections from each mouse were examined from three mice per group. A quantitative analysis of demyelination was performed using Adobe Photoshop CS5 (Adobe Systems, Mountain View, CA) extended; images at the same magnification were straightened. The area of the entire spinal cord was selected by a lasso tool and measured as number of pixels displaying positive signal. Only regions of demyelination were selected, and then demyelinated area was expressed as % area (demyelinated area/total spinal cord area × 100). The number of infiltrated mononuclear cells was counted manually from images under higher magnification by a blinded observer. The method for quantifying demyelination was modified from McCully et al. [21].

### Detection of ROS

For detection of ROS production after EAE, mice were injected intraperitoneally with 10 mg/kg dihydroethidium (dHEt) (Molecular Probes, Invitrogen) at 21 days after first immunization. The dHEt is preferentially oxidized by superoxide, although it is also sensitive to other reactive species, providing an index of the production of reactive species. After its oxidation in the cytosol, dHEt produces the fluorescent compound ethidium (Et) [22]. Mice were euthanized 3 h after dHEt injection and transcardially perfused with 0.9 % saline followed by 4 % PFA fixation. In 30 μm cryostat sections, Et signals were photographed with a LSM 710 confocal imaging system (Carl Zeiss, Germany) with an excitation of 510–550 nm and an emission greater than 580 nm. Images were taken from the thoracic spinal cord section. Et signal intensity was measured by image J (NCBI, MD) and expressed as mean gray value.

### Immunohistochemical examination of the spinal cord

The spinal cord, previously perfused with 4 % paraformaldehyde, was post-fixed in the same fixative overnight and cryoprotected in PBS containing 30 % sucrose at 4 °C for 2 days. Frozen 30-μm sections of the spinal cord were immunohistochemically stained with specific antibodies against cell surface molecules for cluster differentiation (CD) according to conventional methods. Monoclonal antibodies against CD4 (BD Bioscience, CA), CD8 (BD Bioscience), F4/80 (eBioscience, CA) produced in a rat, or a polyclonal antibody against CD20 produced in goat (SantaCruz Biotechnology, CA) were used as the primary antibodies. A secondary antibody against rat IgG or goat IgG (Vector, Burlingame, CA, USA) was used. The immunoreaction was visualized with 3,3′-diaminobenzidine (DAB) after incubation in ABC reagent (Vector, Burlingame, CA). Finally, they were mounted on 0.5 % gelatin-coated slides, coverslipped, and photographed with an Olympus microscope. To assess nonspecific effects, a few sections in every experiment were incubated in a buffer without primary antibodies. This procedure always resulted in a complete lack of immunoreactivity.

### Reverse transcription polymerase chain reaction analysis

The spinal cord and spleen were perfused with cold PBS, and extraction of total RNA was performed with the NucleoSpin® RNA (Macherey-Nagel, Düren, Germany). Reverse transcription polymerase chain reaction (RT-PCR) was performed to measure the levels of mRNA specific for tumor necrosis factor alpha, interleukin-1 beta, and interleukin 12 in the extract according to the manufacturer’s instructions. First-strand cDNA was synthesized from 1 μg of total RNA using the ReverTra Ace® qPCR RT Master Mix with gDNA Remover (Toyobo Corporation, Osaka, Japan) according to the manufacturer’s instruction. The oligonucleotide primers were as follows: TNF-α forward 5′-TCT CAT CAG TTC TAT GGC CC-3′, reverse 5′-GGG AGT AGA CAA GGT ACA AC-3′; IL-1β forward 5′-TTG ACG GAC CCC AAA AGA TG-3′, reverse 5′-AGA AGG TGC TCA TGT CCT CA-3′; IL-12 forward 5′-CAC GCC TGA AGA AGA TGA CA-3′, reverse 5′-AGT CCC TTT GGT CCA GTG TG-3′; and β-actin forward 5′-TGG AAT CCT GTG GCA TCC ATG AAA C-3′, reverse 5′-TAA AAC GCA GCT CAG TAA CAG TCC G-3′. RT-PCR products were separated on an agarose gel, and then RNA bands were quantified using Image J after staining with ethidium bromide.

### Detection of blood–brain barrier disruption by IgG extravasation

To detect blood–brain barrier disruption, histological analysis of IgG extravasation was performed with the thoracic spinal cord of normal or EAE mice at 21 days after first MOG injection. Free-floating 30 μm coronal sections from the spinal cord were immunostained with biotinylated horse anti-mouse IgG (Vector, Burlingame, CA). From the spinal cord section images, the IgG-stained area was also measured by Image J. Measurement of the IgG-stained area was quantified using the method modified from Tang et al. [23]. Briefly, to quantify the area of IgG leakage, the image was loaded into Image J and converted into an 8-bit image. Then, the image was thresholded using the menu option. The type was set to black and white and the bottom slider set to a value of 106. The resulting thresholded image is binary and will only show the region of IgG leakage. To measure this area, the menu option Analyze → Measure was selected. The selected region of the spinal cord in the whole image was sorted, and then area of IgG leakage was expressed as % area.

### Detection of neutrophil infiltration

To detect neutrophil infiltration in the EAE spinal cord, sections were incubated in rabbit anti-myeloperoxidase (MPO, diluted 1:100, Thermo Fisher Scientific, Waltham, MA) in PBS containing 0.3 % Triton X-100 overnight at 4 °C. After washing three times for 10 min with PBS, sections were incubated in Alexa Fluor 488 donkey anti-rabbit IgG antibody (diluted 1:250, Invitrogen, Grand Island, NY) for 2 h at room temperature. The sections were washed three times for 10 min with PBS and mounted on gelatin-coated slides. All images were captured using a fluorescence microscope (Olympus upright microscope IX70, Olympus, Shinjuku, Tokyo, Japan). To quantify MPO staining results, five sections from each mouse were examined from five mice per group. A blinded observer counted the total number of MPO-positive cells in region of interest.

### Detection of NADPH oxidase activation

To identify p47^phox^ subunit expression in the EAE spinal cord, double immunofluorescence staining was performed. Thoracic spinal cord sections were incubated in mixture of rabbit anti-p47 (diluted 1:200, Novus, USA)/mouse anti-microtubule-associated protein 2 (MAP2, diluted 1:200, Millipore, Billerica, MA) in PBS containing 0.3 % Triton X-100 overnight at 4 °C. After washing three times for 10 min with PBS, sections were incubated in a mixture of Alexa Fluor 488 donkey anti-mouse IgG and Alexa Fluor 594 anti-rabbit IgG antibodies (diluted 1:250, Invitrogen, Grand Island, NY) for 2 h at room temperature. The sections were washed three times for 10 min with PBS and mounted on gelatin-coated slides. All images were captured using a LSM710 confocal microscope (Carl Zeiss, Germany).

### Primary microglia cell culture preparation

Mixed glial cultures were prepared from 1-day-old mouse pups of both sexes in 24-well plates as described previously [24]. At confluence, microglia were harvested by gently shaking and re-plated at a density of 5 × 10^5^ cells/well on a 24-well plate. The microglia cultures were subsequently maintained in glial conditioned medium for 24–48 h after which they were used for experiments. The experimental drugs were all dissolved in Eagles minimal essential medium (MEM). Bovine serum albumin (BSA) was used as a control condition for MOG. The MOG dose of 3 μg/ml was selected from the dose range of 1–10 μg/ml because it was the lowest dose to induce a clear (~50 %) increase in number of morphologically activated microglia, and highest tested doses did not show a significant increase in their efficacy (caused ~61 % of microglia to be morphologically activated). Apocynin was dissolved with DMSO, which resulted in a final concentration of 0.0075 % to be used in experiments. DMSO is well known to act as a free radical scavenger, but at low doses, this effect is negligible (data not shown).

### Analysis of microglial morphology, transformation, and proliferation

Microglial morphology and proliferation was determined by cell assessment and counting in five randomly selected phase contrast microscopic fields per culture well. Values were normalized to counts in control wells from the same 24-well plate. Microglia with two or more thin processes were considered as ramified, resting microglia, and microglia with less than two processes, or with amoeboid cell soma, were classified as activated [25]. The numbers of resting and activated microglia were counted in five randomly selected fields per culture well. Evaluations in this study were performed by an observer blinded to the experimental conditions.

### Detection of superoxide production in vitro

For 5 μM dHEt, detection was performed every 15 min within first hour by plate reader with excitation/emission wavelength of 510/580 nm, respectively. Results are presented as a fold change from base line dHEt readings normalized to control condition (BSA) at the peak time of superoxide production (45–60 min).

### Analysis of cytokine release

Cytokine release was analyzed from culture supernatant collected 24 h after the exposure to MOG by Mouse Inflammation antibody Array C1 (RayBiotech, Inc.), detecting 40 different pro- and anti-inflammatory cytokines according to the manufacturer’s instruction. The cytokine-release data was normalized to protein levels to balance the mild proliferation induced by MOG and prevented by apocynin treatment. The data is presented as a fold change compared to control levels.

### Statistical analysis

EAE clinical scores were reported as average ± sem. GLM repeated measures were conducted to investigate differences of clinical scores changing over time among groups using SPSS ver.21. The fluorescence intensity and T cell proliferation data were expressed as the mean ± sem and analyzed for statistical significance using a one-way ANOVA, followed by a Bonferroni post hoc test. Cell culture data were expressed as the mean ± sem compared with ANOVA followed by the Bonferroni’s test for multiple group comparisons. *p* < 0.05 was considered significant.

## Results

### Apocynin ameliorates clinical signs and disease progression of MOG-induced EAE

**Fig. 1:**
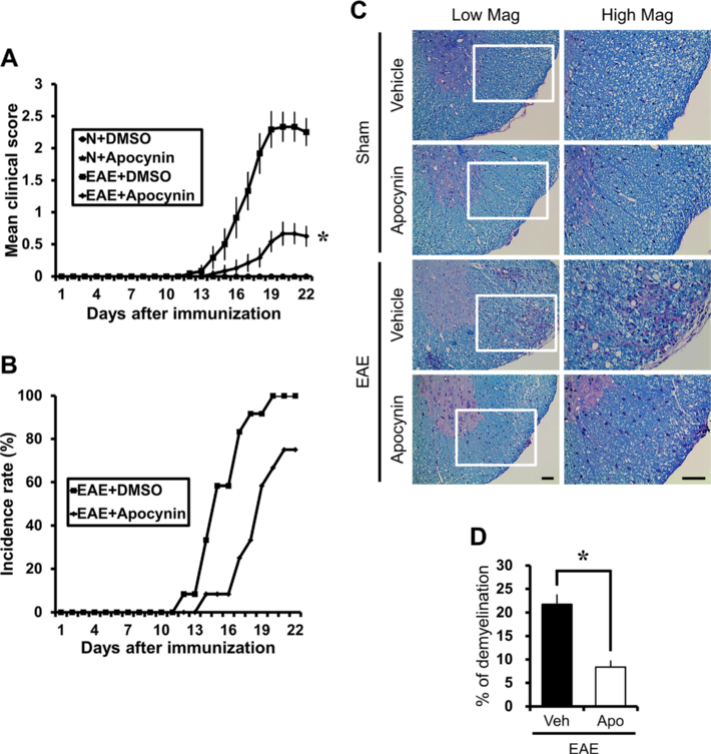
Apocynin ameliorates the clinical signs and disease progression of MOG-induced EAE. Clinical disease scores were recorded daily until 21 days after induction. The clinical signs of EAE first appeared on day 11 and reached peak levels on day 19 from the 1st MOG (M) immunization. Apocynin (EAE + apocynin, *n* = 12) or its vehicle (EAE + vehicle, *n* = 12) was orally administered during the entire period. Control groups also received oral apocynin (sham + apocynin, *n* = 6) or vehicle only (sham + vehicle, *n* = 7) without (N) MOG immunization. **a** Apocynin profoundly reduced the EAE clinical score induced by MOG. Data are mean ± sem (*n* = 6–12). *p < 0.05 compared with apocynin-treated group. General linear models verified statistically significant differences in clinical scores by time and treatment, and interaction between time and treatment was also noticed. **b** Apocynin reduced the incidence rate of EAE induced by MOG. **c**, **d** Apocynin reduced MOG-induced demyelination in spinal cord of EAE mice. Mice were sacrificed 3 weeks after MOG injection, and sections of spinal cord were evaluated for damaged myelin sheaths using luxol fast blue (LFB). The presence of white matter lesions are reflected by reduced LFB staining in the spinal cord. **c** LFB staining of the spinal cord shows extensive demyelination in the MOG-immunized EAE mice. EAE-associated demyelination is almost completely absent in EAE mice treated with apocynin. The *square* area in the low magnification view seen in the left-hand panel was enlarged four hundred times and illustrated in the right-hand panel. *Scale bar* represents 50 μm. **d** The graph represents percent of demyelination of white matter of the thoracic spinal cord with or without apocynin treatment in EAE mice. Data are mean ± sem (*n* = 3). *p < 0.05 compared with the apocynin-treated group

Mice immunized with MOG (35-55) developed severe EAE symptoms with complete hind limb paralysis. As shown in Fig. 1, consecutive administration of apocynin from the beginning of MOG injection resulted in amelioration of the clinical score (Fig. 1a) and incidence rate (Fig. 1b) of EAE. Apocynin treatment decreased the motor deficit score at 19 days from 2.33 ± 0.22 (of 5 maximum) to 0.67 ± 0.18, a 71.2 % reduction (Fig. 1a). General linear models verified statistical significant differences in clinical scores by time (*F* = 23.648, *p* < .001) and treatment (*F* = 28.793, *p* < .001), and interaction between time and treatment was also noticed (*F* = 16.722, *p* < .001).

### Apocynin reduces MOG-induced white matter damage in the spinal cord of EAE mice

LFB staining of the spinal cord revealed extensive demyelination in the MOG-immunized EAE mice (Fig. 1c). In contrast, apocynin-treated EAE mice showed much less demyelination. Compared with the vehicle-treated EAE group, the apocynin-treated EAE group had a 61.6 % reduction in demyelination in the examined spinal cord sections (Fig. 1d).

### MOG-induced ROS production is reduced by apocynin

**Fig. 2:**
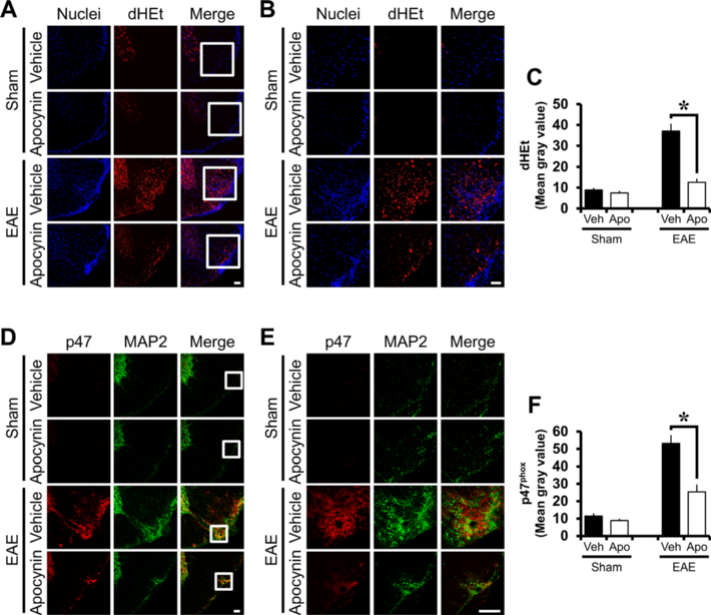
MOG-induced ROS production and p47^phox^ expression are reduced by apocynin. ROS production in the spinal cord was detected by dHEt fluorescence staining 21 days after the first immunization. Mice were sacrificed 3 h after dHEt injection. **a** EAE mice (EAE + vehicle, *n* = 3) showed substantially increased Et fluorescence intensity in the white matter of the spinal cord. Apocynin (EAE + apocynin, *n* = 3) treatment showed substantial reduction of this Et fluorescence intensity in EAE mice. **b** The *square* area in the low magnification of panel **a** was enlarged four hundred times and illustrated in the right-hand panel. *Scale bar* represents 50 μm. **c** The graph represents Et fluorescence intensity as mean gray value (Image J). Data are mean ± sem (*n* = 3). *p < 0.05 compared with the apocynin-treated group. **d** To test whether apocynin treatment influences NADPH oxidase activation in EAE mice, NADPH oxidase subunit (p47^phox^) and neurons (MAP2) were immunohistochemically stained. Sham-operated spinal cord section showed only weak p47^phox^ expression. A prominent increase in p47^phox^ expression was seen throughout the spinal cord 21 days after MOG injections (EAE + vehicle, *n* = 3). However, the expression of p47^phox^ was reduced by apocynin treatment (EAE + apocynin, *n* = 3) in EAE mice. **e** The *square* area in the low magnification of **d** panel was enlarged four hundred times and illustrated in the right-hand panel. *Scale bar* represents 50 μm. **f** The graph represents p47^phox^ fluorescence intensity as mean gray value (Image J). Data are mean ± sem (*n* = 3). *p < 0.05 compared with apocynin-treated group

To test whether ROS production occurred after MOG immunization in the spinal cord, mice were injected with dHEt at 21 days after first immunization and then spinal cords were harvested 3 h after dHEt injection. ROS production was detected in the thoracic spinal cord by Et signal. Sham-operated mice represented less Et fluorescence intensity. MOG-immunized EAE mice showed remarkable increase of Et signal in the white matter of the spinal cord (Fig. 2a, b). However, apocynin-treated EAE mice showed reduced Et fluorescence intensity. Compared with the vehicle-treated EAE group, the apocynin-treated EAE group had a 66.17 % reduction in ROS production in the examined spinal cord sections (Fig. 2c).

### MOG-induced NADPH oxidase subunit expression is reduced by apocynin

To test whether apocynin treatment influences NADPH oxidase activation in EAE mice, we used double immunofluorescence (Fig. 2d, e). Compared to sham-operated control, the spinal cord of MOG-immunized EAE group showed increased p47^phox^ intensity. Apocynin treatment decreased p47^phox^ fluorescence intensity in EAE mice, which represents inhibition of NADPH oxidase activation in EAE mice.

### Decreased mononuclear cell infiltration in apocynin-treated EAE mice

**Fig. 3:**
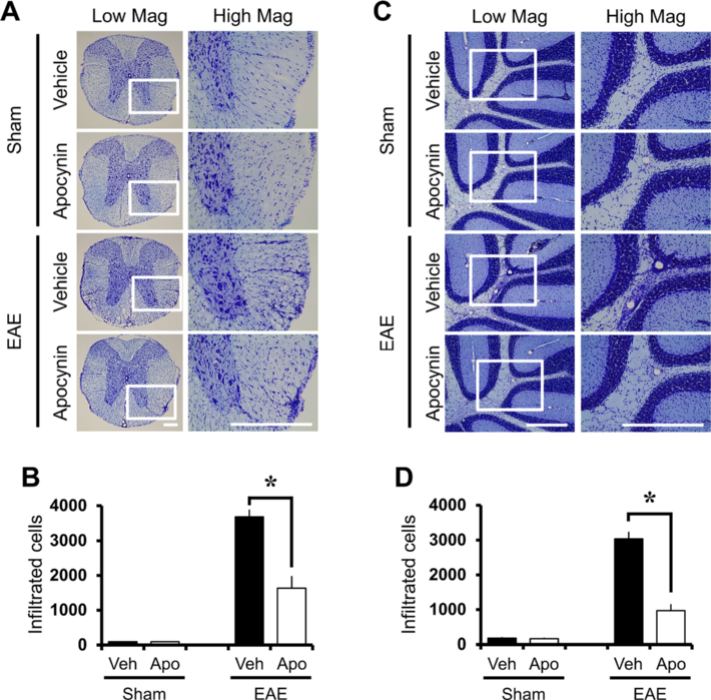
Apocynin treatment attenuates MOG-induced mononuclear cell infiltration into white matter of spinal cord and cerebellum in mice. Three weeks after the initial immunization of MOG, infiltration of mononuclear cells around small vessels in the spinal cord and cerebellum was detected with cresyl violet staining. EAE mice (EAE + vehicle, *n* = 3) revealed intensive infiltration of mononuclear cells around the white matter of the spinal cord (**a**) and cerebellum (**c**). However, apocynin treatment (EAE + apocynin, *n* = 3) reduced mononuclear cell infiltration into the white matter of the spinal cord. *Scale bar* represents 200 μm. The graphs represent number of infiltrated cells in the white matter of thoracic spinal cord (**b**) and cerebellum (**d**) with or without apocynin treatment in sham-operated and EAE mice. Data are mean ± sem (*n* = 3). *p < 0.05 compared with apocynin-treated group

EAE mice displayed intensive infiltration of mononuclear cells around the white matter of the spinal cord (Fig. 3a, b) and cerebellum (Fig. 3c, d). Results presented in Fig. 3 show markedly reduced infiltration of mononuclear cells in sections of the spinal cord and cerebellum derived from apocynin-treated EAE mice compared with vehicle-treated EAE mice.

### Apocynin modulates microglial responses to MOG

**Fig. 4:**
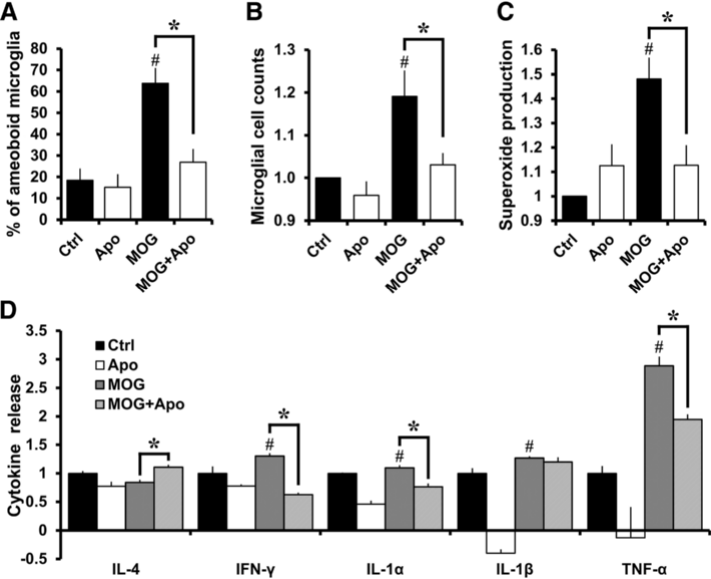
Apocynin modulates microglial responses to MOG. MOG (3 μg/ml) induced morphological transformation of microglia towards amoeboid morphology (**a**) and increased cell counts (**b**) suggesting proliferation within 24 h from stimulation. **c** The superoxide production was measured within an hour after the MOG stimuli. Apocynin (Apo, 600 μM) treatment prevented these microglial responses to MOG stimulation and also reduced basal response levels. Bovine serum albumin (3 μg/ml) was utilized as a control (Ctrl). The graphs show the quantitation of the data. The units shown in B and C are reported as fold changes compared to Ctrl. Data are mean ± sem (*n* = 4-5) *p < 0.05, #*p* < 0.05 compared with Ctrl group. **d** Cytokine analysis demonstrate that MOG increases release of pro-inflammatory cytokines such as IFNɣ, IL-1α, IL-1β, and TNFα and reduces release of anti-inflammatory cytokine such as IL-4, while apocynin treatment reduced pro-inflammatory cytokine release and promoted anti-inflammatory IL-4 release. The units are reported as fold changes compared to Ctrl. Data are mean ± sem (*n* = 3) *p < 0.05, #*p* < 0.05 compared with Ctrl group

The effects of apocynin in EAE mice were further evaluated in primary microglia cultures stimulated with MOG. Marked increase in transformation towards amoeboid morphology, proliferation, superoxide production, and pro-inflammatory cytokine release (IFNɣ, IL-1α, IL-1β, and TNFα) was induced by 3 μg/ml MOG. MOG induced a significant increase in the release of pro-inflammatory cytokines. Apocynin prevented these effects of MOG and sifted cytokine release pattern towards anti-inflammatory phenotype by significantly promoting IL-4 release and decreasing pro-inflammatory cytokines (Fig. 4a–d).

### Apocynin suppresses T cell, B-lymphocyte, and microglial infiltration in EAE mice

**Fig. 5:**
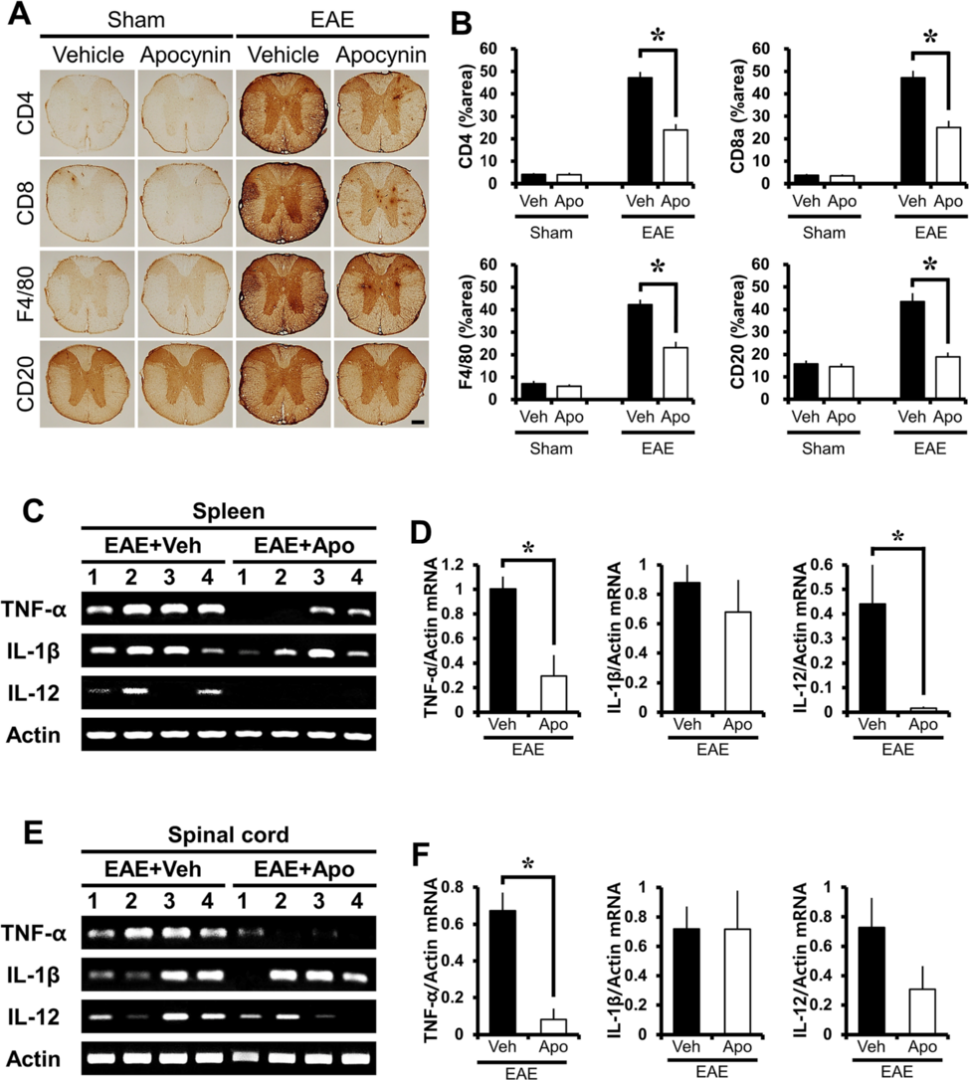
Apocynin treatment attenuates MOG-induced immune cell infiltration in the spinal cord and production of pro-inflammatory cytokines in the spleen and spinal cord. **a** PFA-fixed sections of the thoracic spinal cord were immunohistochemically stained with antibodies against cell surface molecules such as CD4, CD8, F4/80, and CD20. Immunostaining revealed that T cell, B cell, and microglia/macrophage-labeled cells were extensively infiltrated into the white matter of the spinal cord in EAE mice (EAE + vehicle, *n* = 3). However, apocynin treatment reduced the infiltration of immune cells into the white matter of the spinal cords in EAE mice (EAE + apocynin, *n* = 3). *Scale bar* represents 200 μm. **b** The graphs represent percent area of CD4, CD8, F4/80, and CD20 immunoreactivity in the white matter of thoracic spinal cord with or without apocynin treatment in sham-operated and EAE mice. Data are mean ± sem (*n* = 3). *p < 0.05 compared with apocynin-treated group. Spleen and spinal cord perfused with cold PBS were used for extraction of total RNA. Expression of mRNA was determined with the RT-PCR method. RT-PCR results demonstrated an increase of TNF-α and IL-12 in the spleen (**c**) and spinal cord (**e**) of EAE mice (EAE + vehicle, *n* = 4). However, apocynin (EAE + apocynin, *n* = 4) significantly reduced the EAE-associated increase in mRNA expression of TNF-α and IL-12. The graphs represent quantification of TNF-α and IL-12 expression with or without apocynin treatment in the spleen (**d**) and spinal cord (**f**) of EAE mice. Data are mean ± sem (*n* = 4). *p < 0.05 compared with apocynin-treated group

The above results were further confirmed by immunohistochemical staining for CD4+ T, CD8+ T, and CD20+ B lymphocytes. Spinal cords derived from EAE mice were rich in CD4+ T, CD8+ T, and CD20+ B cell infiltrates, while treatment with apocynin significantly decreased CD4+ T, CD8+ T, and CD20+ B cell infiltrates into the white matter. F4/80 expression was apparently increased on day 21 post-MOG immunization. In contrast, a significantly reduced number of F4/80 stained cells were present after apocynin treatment (Fig. 5a, b).

### Apocynin suppresses production of pro-inflammatory cytokines in the spleen and spinal cord of EAE mice

To test whether apocynin treatment influences production of pro-inflammatory cytokines in EAE mice, we measured the levels of mRNA specific for tumor necrosis factor alpha, interleukin-1 beta, and interleukin 12 in the spleen and spinal cord. RT-PCR analysis exhibited that mRNA of TNF-α, IL-12, and IL-1β were highly upregulated in the spleen and spinal cord of EAE mice. Apocynin-treated EAE mice showed significantly inhibited production of TNF-α and IL-12 mRNA in the spleen and TNF-α mRNA in the spinal cord. However, IL-1β mRNA was not seen to differ between vehicle- and apocynin-treated groups in the spleen and spinal cord of EAE mice (Fig. 5c–f).

### Apocynin reduces MOG-induced blood–brain barrier disruption

**Fig. 6:**
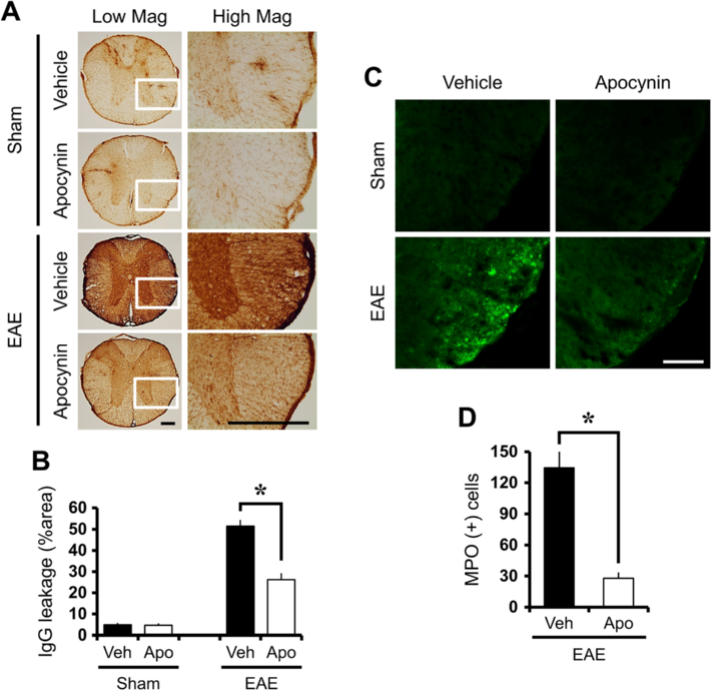
Apocynin reduces blood–brain barrier disruption and neutrophil infiltration in the white matter of EAE spinal cord. **a** Images represent immunohistochemical characterization of IgG extravasation in the thoracic spinal cord of EAE mice. Sham-operated spinal cord section showed only weak IgG immunoreactivity (IR) primarily confined to the BBB-deficient area. Some perivascular extravasation of IgG (*brown color*) was detected in the thoracic spinal cord of normal mice. Prominent extravasation of IgG is seen throughout the parenchyma of the spinal cord 21 days after MOG injections (EAE + vehicle, *n* = 3). However, the extensive diffusion of IgG IR was reduced by apocynin treatment in EAE mice (EAE + apocynin, *n* = 3). *Scale bar* represents 200 μm. **b** The graph represents percent area of IgG IR in the white matter of thoracic spinal cord with or without apocynin treatment in sham-operated and EAE mice. Data are mean ± sem (*n* = 3). *p < 0.05 compared with apocynin-treated group. **c** Fluorescence images display neutrophil infiltration by myeloperoxidase (MPO) staining in the spinal cord. EAE mice (EAE + vehicle, *n* = 5) revealed intensive infiltration of neutrophil in the white matter of the spinal cord. However, apocynin treatment reduced neutrophil infiltration into the white matter of spinal cord in EAE mice (EAE + apocynin, *n* = 5). *Scale bar* represents 100 μm. **d** The bar graph shows the quantification of MPO(+) cells in the spinal cord. The number of MPO(+) cells is significantly different between vehicle (Veh)- and apocynin (Apo)-treated group in the EAE mice. Data are mean ± sem (*n* = 5). *p < 0.05 compared with apocynin-treated group

To evaluate the putative damage of the BBB, we looked for leakage of serum IgGs using immunohistochemistry. Compared with sham-operated control, there was a significant increase in IgG extravasation observed in the thoracic spinal cord at 21 days after first MOG immunization. Compared to the vehicle-treated EAE mice, apocynin-treated EAE mice showed significantly reduced IgG extravasation (Fig. 6a, b).

### Decreased MPO-positive cell infiltration in apocynin-treated EAE mice

To test whether neutrophil infiltration occurred after MOG immunization, spinal cord sections were measured by myeloperoxidase (MPO) infiltration [26, 27]. MPO is a heme protein synthesized during myeloid differentiation that constitutes the major component of neutrophil azurophilic granules [28]. While MPO is predominantly released by neutrophils, monocyte lineage cells are also capable of secreting MPO [29]. EAE mice showed intensive infiltration of neutrophils around the white matter of spinal cord (Fig. 6c, d). Results presented show markedly reduced infiltration of neutrophils in the spinal cord derived from apocynin-treated EAE mice compared with vehicle-treated EAE mice.

### Long-term protective effects of apocynin after EAE

**Fig. 7:**
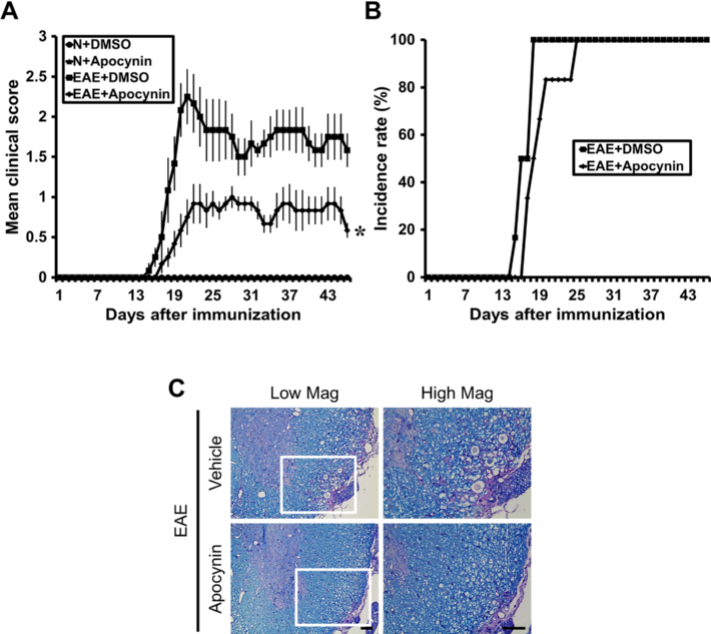
Long-term protective effects of apocynin after EAE induction. Clinical disease scores were recorded daily until 45 days after induction. The clinical signs of EAE first appeared on day 14 and reached peak levels on day 20 from the 1st MOG (M) immunization. Apocynin (EAE + apocynin, *n* = 6) or its vehicle (EAE + vehicle, *n* = 6) was orally administered during the entire period. Control groups also received oral Apocynin (sham + apocynin, *n* = 2) or vehicle only (sham + vehicle, *n* = 2) without (N) MOG immunization. **a** Apocynin profoundly reduced the EAE clinical score induced by MOG. Data are mean ± sem (*n* = 2–6). *p < 0.05 compared with apocynin-treated group. General linear models verified statistical significant differences in clinical scores by time and treatment and interaction between time and treatment was also noticed. **b** Apocynin did not influence the rate of EAE induction by MOG. **c** LFB staining of the spinal cord shows extensive demyelination in the MOG-immunized EAE mice. EAE-associated demyelination is almost completely absent in EAE mice treated with apocynin. The *square* area in the low magnification of the left-hand panel was enlarged two times and illustrated in the right-hand panel. *Scale bar* represents 50 μm

To test whether chronic apocynin treatment is also effective against EAE disease progression, apocynin was administered by gavage once per day for 45 days after the first immunization with MOG. As shown in the short-term treatment experiments, chronic treatment with apocynin also reduced the clinical score and histological findings. As shown in Fig. 1, long-treatment with apocynin also produced improvement in the clinical score and reduced demyelination in the EAE animals. Apocynin treatment decreased the motor deficit score at 20 days from 2.3 ± 0.34 (of 5 maximum) to 0.8 ± 0.21, a 66.7 % reduction (Fig. 7a). General linear models verified statistically significant differences in clinical scores by time (*F* = 10.482, *p* < .001) and treatment assignment (*F* = 13.750, *p* < .001), and interaction between time and treatment assignment was also noticed (*F* = 5.511, *p* < .001). And apocynin treatment also decreased demyelination (Fig. 7c). Furthermore, this preservation of the clinical score was evident until at least 45 days after first MOG immunization.

### Post-treatment of apocynin also decreases clinical signs and histological progress of EAE

**Fig. 8:**
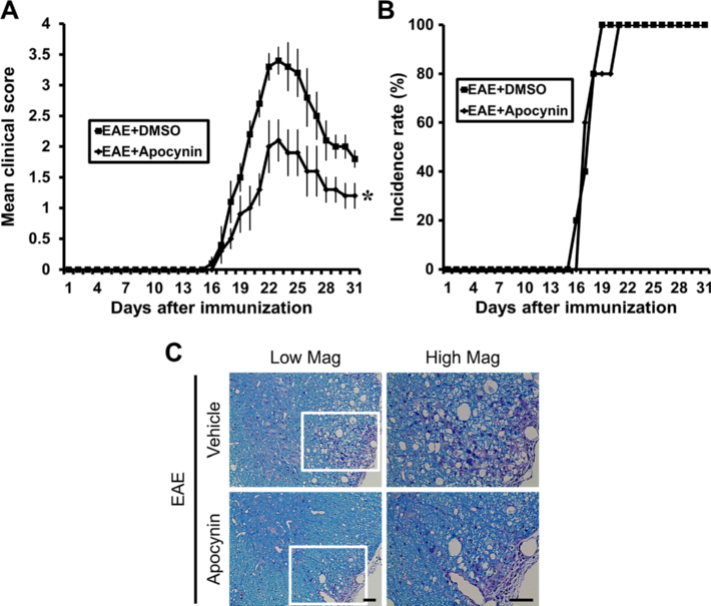
Post-treatment of apocynin also ameliorates the clinical signs and disease progression of EAE. Clinical disease scores were recorded daily until 30 days after induction. Clinical signs of EAE first appeared on day 15 and reached peak levels on day 22 from the 1st MOG (M) immunization. Apocynin (EAE + apocynin, *n* = 5) or its vehicle (EAE + vehicle, *n* = 5) was administered orally from day 18 from the 1st MOG (M) immunization. **a** Post-treatment of apocynin profoundly reduced the EAE clinical score induced by MOG. Data are mean ± sem (*n* = 5). *p < 0.05 compared with apocynin-treated group. General linear models verified statistical significant differences in clinical scores by time and treatment and interaction between time and treatment was also noticed. **b** Post-treatment of apocynin did not exert the incidence rate of EAE induced by MOG. **c** Post-treatment of apocynin reduced MOG-induced demyelination in the spinal cord of EAE mice. Mice were sacrificed 30 days after MOG injection and sections of spinal cord were evaluated for damaged myelin sheaths using luxol fast blue (LFB). White matter lesion was reflected by reduced LFB staining in the spinal cord. LFB staining of the spinal cord shows extensive demyelination in the MOG-immunized EAE mice. EAE-associated demyelination was significantly reduced in EAE mice treated with apocynin. *Scale bar* represents 50 μm

To test whether post-treatment of apocynin is also effective on EAE disease progression, apocynin was delivered 3 days after the typical onset of clinical symptoms (18 days after MOG injection). As shown in the above experiments, post-treatment of apocynin also reduced the clinical score and histological findings. As shown in Fig. 1, post-treatment with apocynin also produced improvement in the clinical score and reduced demyelination of EAE animals. Apocynin treatment decreased the motor deficit score at 22 days from 3.4 ± 0.21 (of 5 maximum) to 2.1 ± 0.33, a 38.24 % reduction (Fig. 8a). General linear models verified statistically significant differences in clinical scores by time (*F* = 54.879, *p* < .001) and treatment assignment (*F* = 12.249, *p* = .008), and interaction between time and treatment assignment was also noticed (*F* = 3.824, *p* < .001). Apocynin treatment also decreased demyelination (Fig. 8c). Furthermore, this preservation of the clinical score was evident until at least 30 days after the first MOG immunization.

## Discussion

The present study shows that oral administration of apocynin, an NADPH oxidase assembly inhibitor, ameliorated clinical signs of MOG-induced EAE. The attenuation of EAE pathogenesis by apocynin was accompanied by reduced white matter damage, ROS production, immune cell infiltration, and BBB disruption.

ROS are involved in the several pathogenic processes of EAE and MS [2, 3, 30]. ROS scavenging has been shown to decrease the severity of clinical symptoms in EAE [2, 31, 32]. ROS damage endothelial cells of the blood–brain barrier [33] and the myelin sheath [34, 35].

Several enzymes are capable of producing ROS, including xanthine oxidase and NADPH oxidase. The cells responsible for production of ROS are believed to be macrophages or activated microglia in the central nervous system [32, 36, 37]. NADPH oxidase is a multi-component enzyme consisting of several subunits that coalesce at the cell membrane upon activation. NADPH oxidase is a membrane-bound electron transfer chain involving a flavocytochrome. Activation of the NADPH oxidase complex is induced by a conformational change in the flavocytochrome, which serves as a switch. Apocynin inhibits the assembly of the cytosol and membrane-bound components of the NADPH oxidase complex [10].

Apocynin is a cell permeable selective inhibitor of NADPH oxidase [38] and has an approximately 1 h half-life [39]. Apocynin has very low toxicity (LD50: 9 g/kg upon oral administration to mice) [11], and it has been demonstrated in past experiments that apocynin is BBB permeable [40, 41]. Therefore, apocynin can affect both compartments as ingested apocynin enters circulation, can affect peripheral T cells, and also penetrates into the brain. Thus, it is conceivable that apocynin has global effects as it accesses both areas. A recent study also demonstrated that apocynin can promote BBB integrity [42]. We delivered apocynin for entire experimental period to prevent MOG-induced NADPH oxidase activation. We believe that continuous administration of apocynin may not be toxic and has a protective effect on MOG-induced ROS production. The present study demonstrates that apocynin not only decreased ROS production but also reduced the number of infiltrating cells, which were identified as CD4, CD8, CD20, and F4/80-immunopositive cells, representing T, B, and macrophage/microglial cells, respectively. This point of view is supported by previous finding that apocynin suppressed the cytokine production of CD8^+^ T cells [43]. Thus, the present study shows that NADPH oxidase activation and subsequent ROS are significant contributors to myelin sheath damage in EAE mice.

Although we found that oral administration of apocynin showed protective effects on EAE pathogenesis, the precise mechanism is still unclear. Previously, we showed that EAE-induced white matter injury and behavioral deficits were caused by abnormal zinc modulation. Excessive extracellular zinc release and intracellular zinc accumulation may cause a sequence of events such as MMP-9 activation, BBB disruption, immune cell infiltration, and autophagy induction. Systemic administration of a zinc chelator (or zinc ionophore), clioquinol, reduced MMP-9 activation, BBB disruption, immune cell infiltration, and myelin destruction. Therefore, we suggested that modulation of extracellular zinc release during EAE is involved in myelin damage in spinal cord white matter [44]. However, our previous study did not elucidate how extracellular zinc causes this sequence of events involved in myelin damage. Zinc alone induced NADPH oxidase activation in neurons. Our previous study showed p47^phox^ and p67^phox^ subunit translocation from the cytoplasm to the plasma membrane after glucose deprivation/glucose reperfusion, and that this translocation is prevented by zinc chelation [5]. Zinc-induced neuronal NADPH oxidase activation in neuronal cultures has also been demonstrated by other groups [45, 46]. Zinc-induced ROS production was also blocked by the hexose monophosphate shunt inhibitor, 6-aminonicotinamide (6-AN), which limits the rate at which NADPH can be formed [47]. Thus, the above studies suggested that zinc release may be an intermediary event in NADPH oxidase activation-induced ROS production in several CNS injuries.

Activated microglia in the CNS has been suggested in the pathogenesis of white matter disorders. Li et al. presented a study that peroxynitrite produced by iNOS and NADPH oxidase in activated microglia may play an important role in the pathogenesis of white matter disorders [48]. Liu et al. demonstrated that blocking the assembly of the NADPH oxidase complex or knocking down p47^phox^ reduced ROS production and attenuated neuroinflammation using isolated myelin and primary microglia culture [49]. However, no studies have previously been performed to evaluate the protective effects of apocynin on EAE-induced white matter damage using an animal model. Our in vitro studies also demonstrated that MOG induces superoxide production within the first hour that can be markedly reduced by apocynin. Apocynin also reduced MOG-induced microglial morphology transformation, proliferation, and pro-inflammatory cytokine release, while promoted IL-4 release. It is quite possible that the initial superoxide production initiated the changes in microglial cytokine expression and release profile, particularly because NADPH oxidase-derived superoxide production occurs hours before changes in cytokines or in other protein expression patterns occur. In fact, the importance of superoxide as a mediator of macrophage phenotype shift from M1 to M2, as well as promoting autoreactive T cell maturation has been demonstrated by utilizing diabetic mice with genetic ablation in NADPH oxidase activity (Nfc1) [50, 51]. Of note, MOG failed to induce NO release, which is a typical pro-inflammatory response, and reflects the fact that microglial responses are very much stimuli dependent. For instance, the pro-inflammatory cytokine TNF-α fails to induce NO production, while LPS does induce prominent NO release [24].

Taken together, EAE-induced white matter damage is caused by a sequence of events such as by aberrant zinc modulation, NADPH oxidase activation, ROS production, and inflammatory chain reaction. In the present study, we first demonstrate that apocynin ameliorates clinical signs of MOG-induced EAE in mice. This amelioration was accompanied by inhibition of the EAE-associated demyelination and infiltration of encephalitogenic immune cells including T cells, B cells, and microglial activation in the spinal cord. Apocynin treatment not only reduced ROS production and BBB breakdown but also reduced inflammatory chain reaction, in which NADPH oxidase activation might be one of the initial steps of EAE-induced myelin damage. Furthermore, the present study found that post-treatment of apocynin also decreased the clinical course of EAE and magnitude of spinal cord demyelination. However, these results must be interpreted with some caution as depletion of the Ncf-1 gene, which leads to a complete loss of NADPH oxidase activity, is seen to actually enhance EAE [52]. The difference might be due to the developmental issues lifelong deletion of NADPH oxidase activity can cause compared to the short time pharmacological inhibition of the enzymatic activity utilized in the present study. Therefore, treatment of multiple sclerosis via inhibition of NADPH activation needs to be pursued with careful consideration for the potentially adverse effects that may arise from complete and sustained cessation of this pathway.

## Conclusions

Based on the present study, our results suggest that NADPH oxidase activation is involved in several steps of MS pathogenesis. The amelioration of EAE progression provided by apocynin suggests that inhibition of NADPH oxidase activation has a high therapeutic potential to treat multiple sclerosis.
